# Familial Hypercholesterolemia Detection Through Machine Learning Algorithms: A Low-Hanging Fruit

**DOI:** 10.1016/j.jacadv.2024.101181

**Published:** 2024-08-21

**Authors:** Laurens F. Reeskamp, Maxim E. Annink, Willemijn A.M. Schonck

**Affiliations:** a Department of Vascular Medicine, Amsterdam Cardiovascular Sciences, Amsterdam UMC, University of Amsterdam, Amsterdam, the Netherlands; b Department of Internal Medicine, OLVG Oost, Amsterdam, the Netherlands

**Keywords:** cardiovascular risk, familial hypercholesterolemia, machine learning

**Figure:**
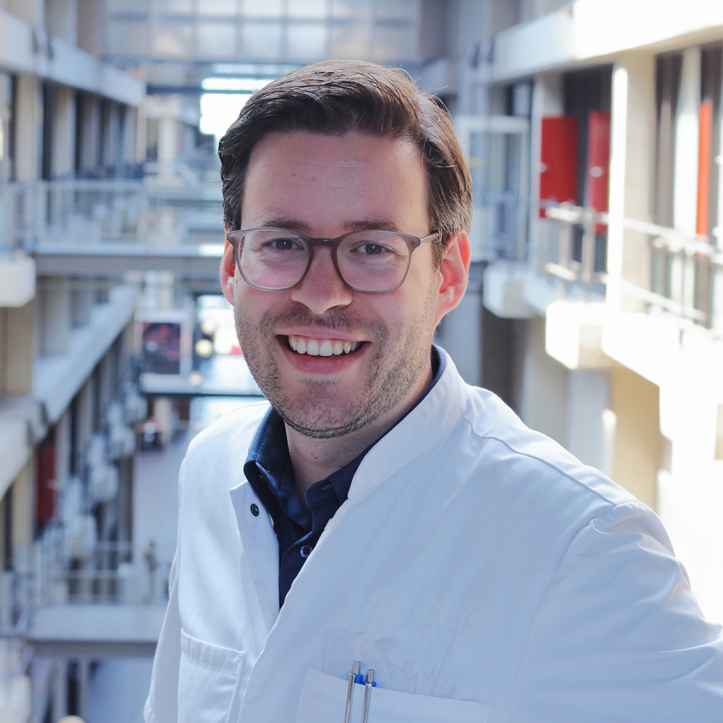

Familial hypercholesterolemia (FH) is a genetic disorder characterized by markedly elevated low-density lipoprotein cholesterol (LDL-C) levels, which substantially increases the risk of premature cardiovascular disease.^1^^,^^2^ Recent studies have demonstrated that the prevalence of FH in the general population is approximately 1:300, with a 23-fold higher prevalence among persons with severe hypercholesterolemia.^3^ Effective lipid-lowering therapies, such as statins, ezetimibe, and proprotein convertase subtilisin/kexin 9 inhibitors, are readily available and initiation of these at a young age can greatly reduce the risk of atherosclerotic cardiovascular disease.^4^

Despite its high prevalence, associated serious cardiovascular sequelae, and available preventive therapies, FH remains underdiagnosed and undertreated. Current estimates suggest that as many as 90% of FH patients worldwide remain unidentified, and more than one-third may not be diagnosed until their first cardiovascular event has already occurred.^5^^,^^6^ Equally concerning are the recent findings from a large global FH registry, which revealed that no more than 3% of patients with FH reach the LDL-C targets recommended by guidelines.^7^ These concerning trends can be attributed to the fact that FH is an asymptomatic disorder until coronary artery disease events manifest, a lack of awareness of FH among physicians, combined with the labor-intensive nature of screening families of FH patients and manually searching existing electronic health records for potential new cases.^8^

As such, novel approaches to address this problem are long overdue. One such approach is the use of machine learning algorithms tailored to the detection of FH. These algorithms employ automated data mining techniques to identify potential new cases of FH within health care systems. They do so by rapidly analyzing electronic health and laboratory records to identify features that are predictive of having yet-to-be-diagnosed FH. An example is the Family Heart Foundation’s flag, identify, network, deliver FH (FIND FH) machine learning model, developed in 2019 and initially trained on electronic health records of clinically diagnosed FH patients from 4 large U.S. health care systems.^9^ Although only 50% of FH patients in this training data set had genetically confirmed FH, its potential value to discover new FH patients was recently demonstrated in a validation cohort, where 34 of the 573 flagged individuals had a (likely) pathogenic FH-causing variant after subsequent genetic validation.^10^

In this issue of *JACC: Advances*, Kim et al^11^ provide further validation of the FIND FH model by applying it to a large U.S. health care system. In brief, the FIND FH algorithm was applied to the electronic health records of 167,955 patients with at least one known cardiovascular risk factor (eg, hypertension or hypercholesterolemia). This resulted in 471 potential FH patients who were automatically flagged by FIND FH and then manually reviewed. Of these, 32 patients had previously established heterozygous FH (10 known homozygous FH patients were excluded beforehand), and 121 patients were considered highly likely to have FH by the clinical reviewers based on elevated LDL-C values and family history. The remaining 318 individuals selected by the algorithm were classified as “suspected FH.” One might question whether this designation is appropriate, as none of these individuals were clinically suspected of having FH by the reviewers.

A key finding of the study is the disparity in medical management between those with established FH diagnoses and those newly identified by the machine learning algorithm. Patients with established diagnoses were more likely to receive lipid panel monitoring, prescriptions for second-line lipid-lowering therapies (eg, ezetimibe and proprotein convertase subtilisin/kexin 9 inhibitors), and specialized cardiovascular evaluations, such as coronary artery calcium testing and lipoprotein(a) measurements. Despite these differences in management, there was no significant difference in the occurrence of major cardiovascular events between the diagnosed and undiagnosed groups.

Thus, integrating machine learning algorithms into routine clinical practice could facilitate the identification of FH patients, enabling timely and targeted interventions that can significantly improve patient outcomes. The algorithm's ability to sift through vast quantities of electronic medical records and flag high-risk individuals represents a substantial advancement in automated technologies for personalized medicine.

However, it should be noted that this study is not without its limitations. First, it appears that validation against the gold standard of FH diagnostics, genetic testing, is lacking in the individuals that were flagged by the model. Instead, clinically unvalidated criteria based on LDL-C values and family history were used. Combined with the fact that there is only a limited overlap between patients identified with phenotypic FH (ie, severe hypercholesterolemia) and genetic FH,^12^^,^^13^ it is difficult to draw conclusions about the performance of this model to identify true, genetically confirmed, FH patients. This problem is likely compounded by the fact that less than half of individuals in the original FIND FH training cohort had a genetic diagnosis to begin with.^9^ Secondly, we concur with the authors that it is unfortunate that the FIND FH model necessitates screened individuals exhibit at least one cardiovascular risk factor. This requirement constrains the model’s utility in identifying young, undiagnosed individuals who do not have other cardiovascular risk factors but would benefit the most from early initiation of lipid-lowering therapy.^14^ Finally, although the FIND FH approach is evidently much less time-consuming than manually screening entire health care systems' populations, it seems inevitable that flagged records undergo rigorous manual review to verify the algorithm's findings. This is a laborious process that might not be easily implemented in many health care systems.

Despite these limitations, the large number of previously undiagnosed potential FH patients identified by the algorithm in the study by Kim et al^11^ highlights a critical gap in FH identification that might be effectively bridged using advanced computational tools. Moving forward, it is essential to validate and assess the algorithm's sensitivity and specificity in other cohorts, as well as run practical implementation studies where such tools run prospectively and in parallel with clinical care. We look forward to follow-up research that builds on these results and foresee that this will improve clinical outcomes through early identification and treatment of FH in a real-world setting.

## Funding support and author disclosures

Dr Reeskamp is cofounder of Lipid Tools; and has received speaker fees from Ultragenyx, Novartis, and Daiichi Sankyo.
